# Eutrophication and macroalgal blooms in temperate and tropical coastal waters: nutrient enrichment experiments with *Ulva* spp.

**DOI:** 10.1111/j.1365-2486.2009.02108.x

**Published:** 2010-09

**Authors:** Mirta Teichberg, Sophia E Fox, Ylva S Olsen, Ivan Valiela, Paulina Martinetto, Oscar Iribarne, Elizabeti Yuriko Muto, Monica A V Petti, Thaïs N Corbisier, Martín Soto-Jiménez, Federico Páez-Osuna, Paula Castro, Helena Freitas, Andreina Zitelli, Massimo Cardinaletti, Davide Tagliapietra

**Affiliations:** * Leibniz-Zentrum für Marine TropenökologieFahrenheitstrasse 6, 28359 Bremen, Germany; † Marine Biological Laboratory, The Ecosystems Center7 MBL St, Woods Hole, MA 02543, USA; ‡ School of Ocean Sciences, University of BangorWales, Menai Bridge, Anglesey LL59 5AB, UK; § Laboratorio de Ecología, Departamento de Biología, Facultad de Ciencias Exactas y Naturales, Universidad Nacional de Mar del PlataCC573 Correo Central, Mar del Plata B7600WAG, Argentina; ¶ Departamento de Oceanografia Biológica, Instituto Oceanográfico, Universidade de São PauloPraça do Oceanográfico 191, 05508-900 São Paulo, SP, Brazil; | Instituto de Ciencias del Mar y Limnología, Universidad Nacional Autónoma de MexicoApartado Postal 811, Mazatlan 82040, Mexico; ** Centre for Functional Ecology, Department of Botany, University of Coimbra3000 Coimbra, Portugal; †† University IUAV of VeniceVenice 30123, Italy; ‡‡ Gruppo VeritasS. Croce 489, Venice 30100, Italy; §§ Consiglio Nazionale delle Ricerche, Istituto di Scienze Marine (CNR-ISMAR)Riva 7 Martiri, 1364/a, 30122 Venice, Italy

**Keywords:** eutrophication, macroalgal growth, N stable isotopes, nitrogen, nutrient limitation, phosphorus, *Ulva*, wastewater

## Abstract

Receiving coastal waters and estuaries are among the most nutrient-enriched environments on earth, and one of the symptoms of the resulting eutrophication is the proliferation of opportunistic, fast-growing marine seaweeds. Here, we used a widespread macroalga often involved in blooms, *Ulva* spp., to investigate how supply of nitrogen (N) and phosphorus (P), the two main potential growth-limiting nutrients, influence macroalgal growth in temperate and tropical coastal waters ranging from low- to high-nutrient supplies. We carried out N and P enrichment field experiments on *Ulva* spp. in seven coastal systems, with one of these systems represented by three different subestuaries, for a total of nine sites. We showed that rate of growth of *Ulva* spp. was directly correlated to annual dissolved inorganic nitrogen (DIN) concentrations, where growth increased with increasing DIN concentration. Internal N pools of macroalgal fronds were also linked to increased DIN supply, and algal growth rates were tightly coupled to these internal N pools. The increases in DIN appeared to be related to greater inputs of wastewater to these coastal waters as indicated by high *δ*^15^N signatures of the algae as DIN increased. N and P enrichment experiments showed that rate of macroalgal growth was controlled by supply of DIN where ambient DIN concentrations were low, and by P where DIN concentrations were higher, regardless of latitude or geographic setting. These results suggest that understanding the basis for macroalgal blooms, and management of these harmful phenomena, will require information as to nutrient sources, and actions to reduce supply of N and P in coastal waters concerned.

## Introduction

**Fig. 1:**
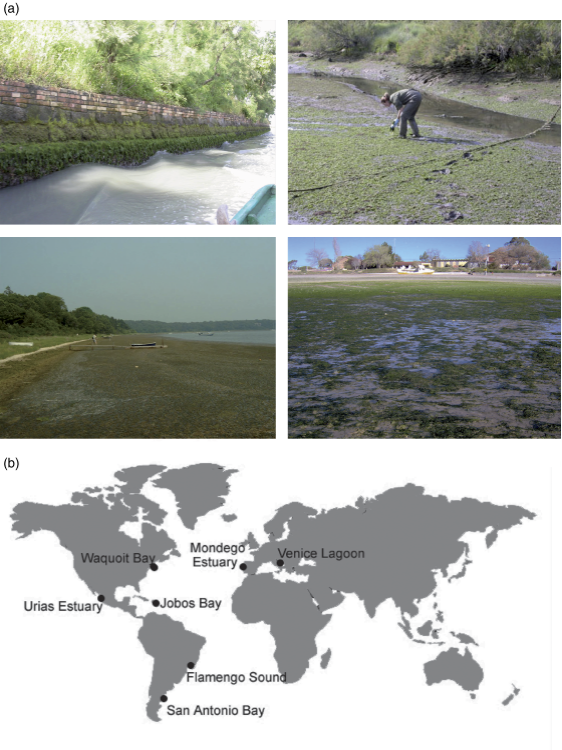
Macroalgal blooms commonly found worldwide. (a) Algal blooms attached to the canal walls of Venice Lagoon, Italy (top, left), on the mudflats of the Mondego Estuary, Portugal (top, right), accumulated wrack and floating mats along shore of Waquoit Bay, MAs, USA (bottom, left), and along San Antonio Bay, Argentina (bottom, right). (b) Map of the experimental sites. Sites include Waquoit Bay, USA, Venice Lagoon, Italy, Mondego Estuary, Portugal, Urias Estuary, Mexico, Jobos Bay, Puerto Rico, Flamengo Sound, Brazil, and San Antonio Bay, Argentina.

Nutrient inputs to coastal waters have increased in coastal environments worldwide as a direct consequence of the growing human population and increased settlement and use of coastal areas (Nixon *et al.*, 1986; Valiela, 2006). These changes in nutrient availability lead to increased eutrophication, a growing threat facing coastal ecosystems (National Research Council (NRC), 2000; Bricker *et al.*, 2008). One common symptom of eutrophication is profuse blooms of marine seaweeds, or macroalgae (Lavery *et al.*, 1991; Sfriso *et al.*, 1992; Valiela *et al.*, 1997; Morand & Merceron, 2005; Fox *et al.*, 2008; Fig. 1a), a feature that has received wide press and public notice (e.g. *New York Times*, July 1, 2008; *Naples Daily News*, March 15, 2006; *Boston Globe*, September 27, 2001), and is widespread along the coasts of the world (Table 1; Raffaelli *et al.*, 1998; Morand & Merceron, 2005).

**Table 1:** Examples of macroalgal blooms reported in different parts of the world's coastlines and some of their ecological and economic consequences

Site	Seaweed taxa*	Some effects	References
*North America*			
Nahant Bay, USA	*Pilayella*	Noxious odor, accumulated on beaches, nuisance to swimming and fishing	Wilce *et al.* (1982), Pregnall & Miller (1988)
Waquoit Bay, USA	*Cladophora, Gracilaria, Ulva*	Replaced seagrasses, anoxia, shell- and fin-fish kills	Valiela *et al.* (1997), Hauxwell *et al.* (2001), Fox *et al.* (2008)
Hog Island Bay, USA	*Ulva, Gracilaria, Codium*	Loss of species diversity	Thomsen *et al.* (2006)
San Francisco Bay, United States	*Ulva*	Anoxia, replaced benthic fauna	Fong *et al.* (1996)
Kanehoe Bay, Hawaii	*Dictyosphaeria*	Replaced corals	Smith (1981)
Southeast Florida, USA	*Codium*	Impact coral reefs	Lapointe *et al.* (2005)
Bermuda	*Cladophora, Laurencia, Codium*	Anoxia, reduced benthic diversity and commercial fisheries	Lapointe & O'Connell (1989)
*Europe*			
Laholm Bay, Sweden NW Baltic Sea	*Ulva, Cladophora*	Replaced seagrasses, nuisance to swimming fishing and boating	Baden *et al.* (1990), Rosenberg *et al.* (1990)
Maasholm Bay, Germany	*Ulva, Pilayella*	Replaced native macroalgae, lowered benthic diversity and fishery yield, nuisance to swimming, fishing and boating	Lotze *et al.* (2000), Worm *et al.* (1999)
Mondego Estuary, Portugal	*Ulva*	Replaced seagrasses, reduced benthic diversity	Martins *et al.* (2001), Cardoso *et al.* (2004)
Venice Lagoon, Italy	*Ulva, Gracilaria, Dictyota*	Anoxia, fish kills, nutrient release, phytoplankton blooms	Sfriso *et al.* (1992), Sfriso & Marcomini (1997)
*South America*			
Gulf of California, Mexico	*Ulva, Gracilaria, Cladophora*	Anoxia, loss of species diversity	Ochoa-Izaguirre *et al.* (2002), Piñon-Gimate *et al.* (2008)
Nuevo Gulf, Patagonia	*Ulva, Undaria*	Accumulated on beaches, interferes with recreational uses	Díaz *et al.* (2002), Piriz *et al.* (2003)
*Asia*			
Quingdao, China	*Ulva*	Loss of species diversity, accumulated on beaches and nuisance for recreational activities	Liu *et al.* (2007, 2009)
Seto Inland Sea, Japan	*Ulva*	Replaced seagrasses	Sugimoto *et al.* (2007)
*Australia*			
Peel-Harvey Estuary, Western Australia	*Cladophora, Ulva, Chaetomorpha*	Accumulated on beaches	Lavery *et al.* (1991)
Tuggerah Lakes Estuary, New South Wales	*Ulva*	Replaced seagrasses, reduced benthic diversity	Cummins *et al.* (2004)

* Macroalgal species are listed only by genus, and all reports of *Enteromorpha* are listed here as *Ulva* (Hayden *et al.*, 2003).

Macroalgal blooms have many detrimental effects. Seaweed wrack accumulates along shorelines and produces foul odors (Wilce *et al.*, 1982), deep canopies of seaweeds physically obliterate other coastal life (Hauxwell *et al.*, 2001), and decay of algal organic matter fosters anoxic conditions that lead to fish and shellfish kills (Baden *et al.*, 1990; Valiela *et al.*, 1992; D'Avanzo *et al.*, 1996; Worm *et al.*, 1999; Diaz, 2001). Macroalgal blooms not only make coastal environments increasingly undesirable for human uses and threaten commercial harvests, but also drastically restructure natural communities and ecosystem function of affected environments (Duarte, 1995; Valiela *et al.*, 1997; Raffaelli *et al.*, 1998; Oesterling & Pihl, 2001; Fox *et al.*, 2009, in press).

It has been argued that N supply is a main control on macroalgal growth in temperate coastal areas (Nixon & Pilson, 1983; Oviatt *et al.*, 1995; Howarth *et al.*, 2000). In tropical latitudes carbonate sediments derived from coral reefs may sequester phosphate (PO_4_^3−^) and may lead to P limitation of macroalgal growth (Lapointe *et al.*, 1992; McGlathery *et al.*, 1994), but other studies show exceptions to this general pattern (Larned, 1998; Fong *et al.*, 2001; Elser *et al.*, 2007), and much research is still needed to better understand the processes driving coastal eutrophication and strategies for water quality management (NRC, 2000; Smith & Schindler, 2009).

External N supply interacts with internal N pools in macroalgae (Fujita, 1985; Bjornsater & Wheeler, 1990; Fong *et al.*, 2003; Teichberg *et al.*, 2008) to create different growth responses (Fujita, 1985; Pedersen & Borum, 1996; Teichberg *et al.*, 2008). The sources of internal N pools have been assessed with stable isotopic methods (McClelland & Valiela, 1998; Aguiar *et al.*, 2003; Savage & Elmgren, 2004; Cole *et al.*, 2005; Teichberg *et al.*, 2007, 2008). Some macroalgae reflect N isotopic signatures of their source with little fractionation, making them potential indicators of anthropogenic nutrient inputs (Savage & Elmgren, 2004; Deutsch & Voss, 2006; Thornber *et al.*, 2008).

No comprehensive study of algal blooms and algal physiological responses has been done on a global scale across latitudes and oligotrophic to eutrophic conditions to understand nutrient limitation and the potential for macroalgal blooms worldwide. This is particularly important at this time as nutrient additions to estuaries and coasts increase, and eutrophication becomes one of the greatest threats to our coasts and estuaries (NRC, 2000; Howarth, 2008). Macroalgal blooms involve relatively few taxa (Valiela *et al.*, 1997; Morand & Merceron, 2005) that are widely distributed throughout the coasts of the world. In particular, species of *Ulva* can be found in many coastal waters (Table 1), thus providing a useful biological model to make geographical comparisons as to the nutrient controls on growth of bloom-forming macroalgae.

In this study we examined the relationship of macroalgal growth, internal nutrient pools, and natural stable isotopes of *Ulva* spp. to different ambient nutrient supplies and tested whether additional N or P above ambient nutrient regimes increased macroalgal growth, increasing likelihood of blooms. We measured growth responses and nutrient content of fronds of local *Ulva* spp. in each of seven coastal systems, including three subestuaries within one estuarine system, for a total of nine sites where ambient nutrient supplies differed. From these experiments, we compared the relative growth responses across latitudes under oligotrophic to eutrophic conditions and under additional N and P enrichment.

## Methods

### Study sites

We chose coastal systems (Fig. 1) in which the level of ambient nutrient concentrations and algal productivity span the range of levels found in oligotrophic to eutrophic coastal waters. In North America, our work was done in Waquoit Bay, MA, USA (41°5′N and 70°5′W), an estuarine complex with a wet, temperate climate, in which we chose three subestuaries, Sage Lot Pond, Quashnet River, and Childs River, that receive different land-derived nitrogen loads, creating widely differing nutrient concentrations and occurrences of macroalgal blooms (Valiela *et al.*, 1997; Fox *et al.*, 2008). In Europe, we worked in Mondego Estuary, Portugal (40°1′N and 8°5′W), and Palude della Rosa, Venice Lagoon, Italy (45°5′N and 12°4′E), eutrophic sites that are also in wet, temperate climates with high macroalgal biomasses (Flindt *et al.*, 1997; Martins *et al.*, 2001). Venice lagoon is historically one of worst-case scenarios against which to compare all other sites (Sfriso *et al.*, 1992; Flindt *et al.*, 1997; Sfriso & Marcomini, 1997). To represent dry, tropical climates, we worked in Jobos Bay, Puerto Rico (17°9′N and 66°2′W), a site with oligotrophic waters and low macroalgal biomass (Bowen & Valiela, 2008) and Urias estuary, Mexico (23°1′N and 106°2′W), a site with significant nitrogen enrichment and high macroalgal biomass (Ochoa-Izaguirre *et al.*, 2002). Flamengo Sound, Brazil (23°3′S and 45°1′W) represents a site with low nutrient loads and a diverse macroalgal community (Corbisier *et al.*, 2006) in a wet, tropical climate, while San Antonio Bay, Argentina (40°7′S and 64°9′W) is an eutrophic site (P. Martinetto *et al.*, unpublished results) in an arid, temperate climate.

### Experimental layout

To test the impact of ambient nutrient supply on growth of *Ulva* spp., we carried out *in situ* cage experiments in the nine sites. We used *Ulva lactuca* Linnaeus in our experiments in Waquoit Bay, Jobos Bay, Urias Estuary, San Antonio Bay, and Mondego Estuary; *Ulva fasciata* Delile in Flamengo Sound; and *Ulva laetevirens* J. E. Areschoug in Venice Lagoon. All macroalgal fronds were collected from the water near the sediment surface, as either floating or attached forms. Incubations were carried out once during the peak of the growing season in each site, except for in Waquoit Bay subestuaries where incubations were carried out several times throughout the growing season. *Ulva* fronds were incubated in experimental cages in waters at each of the nine sites. The cages were 15 cm × 20 cm × 20 cm and constructed of four acrylic plastic sides to allow for light penetration and two mesh sides with 1 mm mesh openings to allow for horizontal water flow through the units, but also to restrict access to cages by grazers. The four acrylic sides of the cages consisted of two sides of 20 cm × 20 cm and a top and bottom of 20 cm × 15 cm. The two mesh sides were 20 cm × 15 cm. The cages were individually attached to a line 3 m apart. The line was anchored approximately 0.3 m from the sediment surface with concrete blocks. Additionally, the cages each had a buoy attached by a rope to allow the cage to float and rotate freely with the water flow. This design allowed for each cage to be suspended within the macroalgal canopy at 0.1–0.3 m from the sediment surface.

To discern the effect of additional N or P enrichment in coastal waters, we measured *Ulva* spp. growth rates after 10–13 days *in situ* in control cages with no nutrients added and in cages where we experimentally added nitrate (NO_3_^−^) or PO_4_^3−^ to the water within the cages. Experimental cages were randomly distributed under the three nutrient treatments, unenriched controls, added NO_3_^−^, and added PO_4_^3−^, with *n*=4 per treatment.

To deliver N and P to macroalgal fronds in cages, NO_3_^−^ or PO_4_^3−^ additions were prepared by dissolving KNO_3_ or KH_2_PO_4_ in a 3% agar solution, making a 2 m KNO_3_ or 1 m KH_2_PO_4_ solution, and by setting the agar in a perforated PVC pipe at the center of the unit to allow for slow release over the course of the 10–13-day experimental run. *In situ* nutrient delivery methods were previously tested for effectiveness (Teichberg, 2007; Teichberg *et al.*, 2008). We used NO_3_^−^ for our N supply in our enrichments because NO_3_^−^ was by far the greatest contributor to total dissolved inorganic nitrogen (DIN) in most of our sites. Water depth ranged between 0.5 and 9 m among sites, with the highest tidal ranges in Mondego Estuary (up to 3.5 m) and San Antonio Bay (up to 9 m). Cages were submerged at all times in all of the sites, except for Mondego Estuary, where cages were out of the water for short periods during low tide. To measure maximum growth response of *Ulva* spp., we carried out experiments during peak growing season in each site.

### Analysis of water nutrient concentrations

To measure ambient and experimental nutrient concentrations to which *Ulva* spp. fronds were exposed during the incubation period, ambient water and water within the experimental cages was sampled at the beginning, middle, and end of the experiment using a 60 mL syringe and sipping water from a thin tube inserted permanently in the center of each cage without disturbing the cages. Water was filtered through a GF/F glass fiber filter into 60 mL plastic sample bottles at the field site and then frozen. All water samples from each site were sent frozen to the Marine Biological Laboratory (Woods Hole, MA, USA) for analysis. Concentrations of NO_3_^−^, NH_4_^+^, and PO_4_^3−^ were measured with a Lachat AutoAnalyzer (Lachat Instruments, Loveland, CO, USA) using standard colorimetric procedures, and mean concentrations were calculated for each treatment over the course of the incubation. Annual nutrient concentrations for each site were used from other studies (supporting information, Table SA1; Carrer *et al.*, 2000; Azevedo, 2002; Ochoa-Izaguirre *et al.*, 2002; National Oceanic and Atmospheric Administration, 2004; Lillebø*et al.*, 2005; Holmes, 2008, P. Martinetto, unpublished results).

### Response of macroalgae

To assess the growth response of macroalgae to ambient nutrient supply and experimental nutrient enrichment, initial and final wet weights of *Ulva* spp. fronds were measured before and following incubation by blotting algae dry with paper towel. Growth rates were calculated as % growth day^−1^ using a linear growth calculation to compare the relative % change in initial and final biomass within each cage over the course of the incubation period. To assess relationships between *Ulva* spp. growth and annual nutrient concentrations in the water among the sites, we used regression analysis. Data that were not normally distributed were log-transformed before regression analysis.

To test whether there were significant growth responses to N or P enrichment within each site, we used a one-way-anova, and *post hoc* Tukey test. To directly compare macroalgal growth responses to the enriched nutrients among the sites, we needed to standardize the growth rates based on the relative response to the enrichment from the controls within a site. We did this by calculating a relative response to NO_3_^−^ and PO_4_^3−^ by subtracting the mean growth rate of the control treatments from the mean growth rates of the nutrient enrichments within each site, and propagating the error. Then, to assess the relationships between a relative response to NO_3_^−^ and PO_4_^3−^ and ambient nutrient concentrations among the sites we used regression analysis. Data that were not normally distributed were log-transformed before analysis.

To determine internal nutrient pools and N isotopic signatures of fronds, samples of *Ulva* spp. were collected from each site, cleaned, dried at 60 °C, and ground to a fine powder with mortar and pestle. Dried macroalgal tissue was analyzed for total N content, and N stable isotopes at the University of California, Davis, Stable Isotope Facility. Regression analysis was used to assess relationships between *Ulva* spp. N content and N isotopes, and water nutrient concentrations among the sites. Data that were not normally distributed were log-transformed before regression analysis.

## Results

### Analysis of nutrient concentrations

**Fig. 2:**
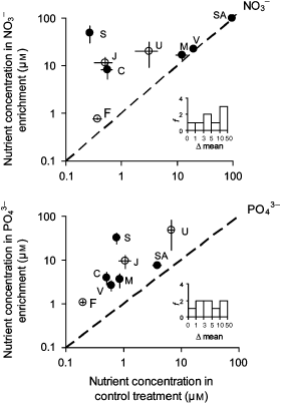
Nutrient concentations (μm) of NO_3_^−^ in the NO_3_^−^ enrichment treatments (top) and PO_4_^3−^ in the PO_4_^3−^ enrichment treatments (bottom) vs. the control treatments. Dashed line represents the 1 : 1 line. Sites include Waquoit Bay, USA (S, C), Venice Lagoon, Italy (V), Mondego Estuary, Portugal (M), Urias Estuary, Mexico (U), Jobos Bay, Puerto Rico (J), Flamengo Sound, Brazil (F), and San Antonio Bay, Argentina (SA). Temperate sites are black symbols and tropical sites are open symbols. Inset histograms represent the frequency of the delta mean, the difference between mean concentrations of NO_3_^−^ and PO_4_^3−^ in the cages that were enriched and corresponding control cages (μm), for all sites.

The concentrations of DIN and PO_4_^3−^ differed substantially among the study sites (Fig. 2). In some cases ambient concentrations measured in this study during the incubation periods differed from the annual nutrient concentrations reported (supporting information, Table SA1). This variation in short-term concentrations from the annual means may be influenced by seasonal differences in nutrient concentrations. The incubations were carried out during the peak of the growing season, and therefore, the high biomass of macroalgae in some of the sites may have rapidly depleted the nutrients from the water column.

The experimental nutrient enrichments of NO_3_^−^ or PO_4_^3−^, both successfully increased nutrient concentrations in the cages above ambient nutrient concentrations during the incubations (Fig. 2), as shown by the points above the 1 : 1 line. Variation in the degree of nutrient enrichment from each site depended on the range in ambient nutrient concentrations as well as the hydrodynamics of each system. For example, the sites with faster flow rates, larger tidal exchange, or wave action (Flamengo Sound, Mondego Estuary, and San Antonio Bay), and sites with higher ambient N concentrations (Mondego Estuary, Venice Lagoon, and San Antonio Bay) showed less of an increase in nutrient concentrations relative to the ambient in spite of the experimental enrichment. To better display the increases with enrichment, we calculated the difference between mean concentrations of NO_3_^−^ and PO_4_^3−^ in the cages that were enriched and corresponding control cages (Fig. 2, insets). In all cases, the data show that treated cages maintained higher concentrations of the enriched nutrient. Concentrations of NO_3_^−^, NH_4_^+^, and PO_4_^3−^ inside control cages were similar to the ambient water concentrations (Teichberg, 2007).

### Response of macroalgae to ambient conditions

**Fig. 3:**
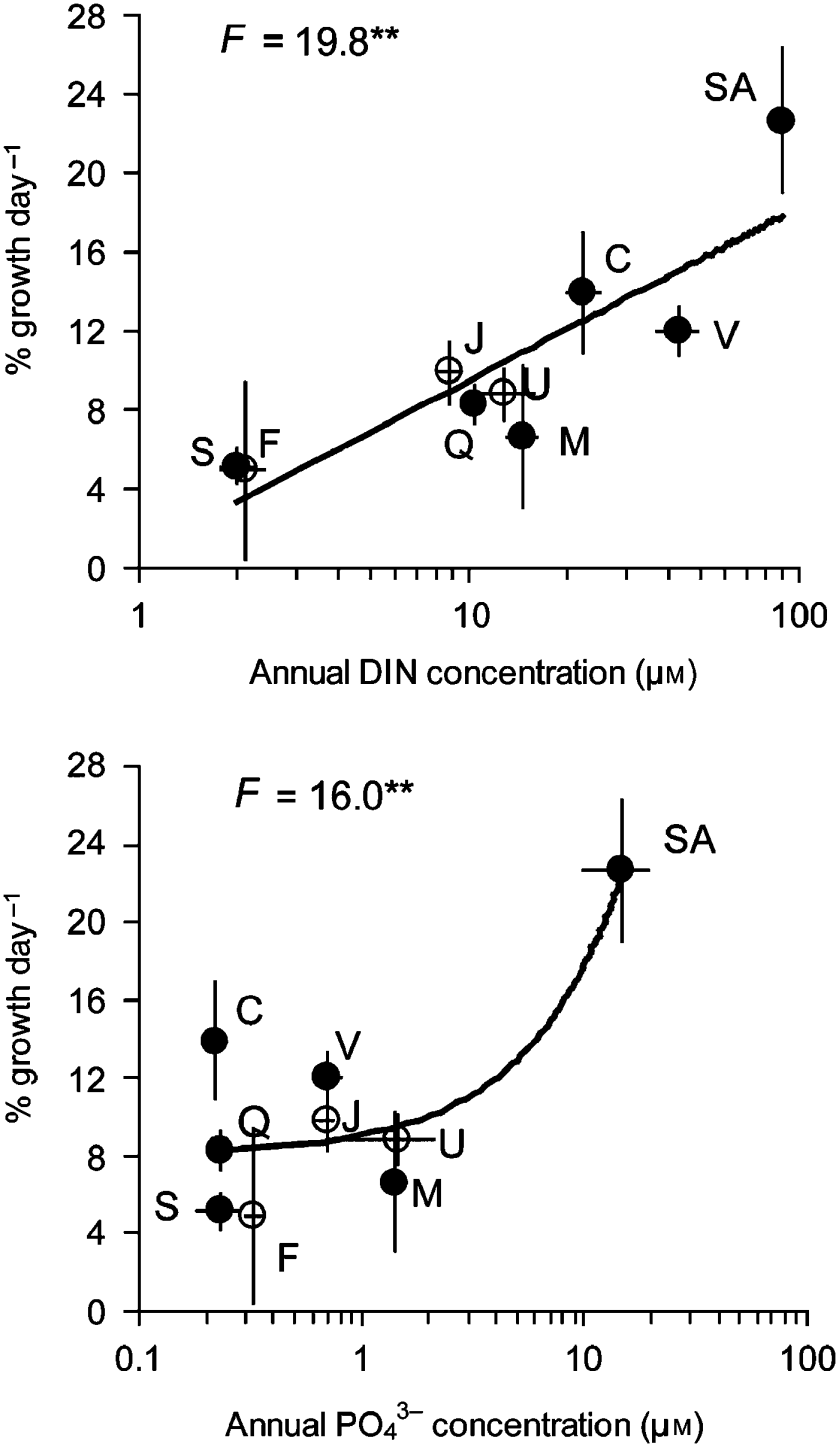
Mean growth rate (% growth day^−1^) of *Ulva* spp. from the control treatments vs. the annual dissolved inorganic nitrogen (DIN, top) and PO_4_^3−^ (bottom) concentrations. Sites include Waquoit Bay, USA (S, Q, C), Venice Lagoon, Italy (V), Mondego Estuary, Portugal (M), Urias Estuary, Mexico (U), Jobos Bay, Puerto Rico (J), Flamengo Sound, Brazil (F), and San Antonio Bay, Argentina (SA). Temperate sites are black symbols and tropical sites are open symbols. For C, Q, S, seasonal means of growth rates are reported. Results of regression analyses are indicated by the *F* values and significance level of ^**^P=0.01.

The growth rates of *Ulva* spp. that were simply incubated with no experimental nutrient addition differed considerably among the sites (Table 2). Rates of growth of fronds of *Ulva* spp. were significantly higher in coastal waters with larger mean annual concentrations of DIN (Fig. 3, top) and PO_4_^3−^ (Fig. 3, bottom). The logarithmic relationship of growth to DIN concentrations was significant (Fig. 3, top), while that of PO_4_^3−^ was driven largely by the high concentration in one site, San Antonio Bay (Fig. 3, bottom). There were no obvious patterns in growth responses in correlation with latitude, with the exception that the highest growth rates were all from temperate systems (Table 2, Fig. 3).

**Table 2:** Comparison of specific growth response of *Ulva* spp. as % growth day^−1^ (mean ± SE) for control, nitrate, and phosphate nutrient treatments during the peak growing season within each site

	% growth day^−1^				
			
Site	Control	NO_3_^−^ added	PO_4_^3−^ added	*F*	*P*
*Waquoit Bay*					
Childs River (C)					
Jun-04	12.6 ± 0.9	–	–		
Sep-04	3.4 ± 0.5	–	–		
Jul-05	25.7 ± 3.1	34.2 ± 9.9	18.8 ± 1.4	1.7	0.25
Quashnet River (Q)					
Jun-04	10.2 ± 0.7	15.9 ± 2.8	12.2 ± 1.3	3.3	0.09
Sep-04	6.3 ± 1.0	–	–		
Sage Lot Pond (S)					
Jun-04	3.8 ± 1.0	–	–		
Sep-04	2.9 ± 0.9	–	–		
Jul-05	8.9 ± 0.2	42.6 ± 7.8	8.0 ± 2.4	20.9	0.001
Jobos Bay (J)	9.9 ± 1.6	23.5 ± 4.4	8.5 ± 1.3	11.8	0.003
Urias Estuary (U)	8.8 ± 1.3	10.8 ± 1.5	9.6 ± 1.8	0.6	0.59
Flamengo Sound (F)	4.9 ± 4.5	4.1 ± 3.8	6.1 ± 1.7	0.1	0.90
San Antonio Bay (SA)	22.6 ± 3.7	19.6 ± 1.3	24.7 ± 2.2	0.4	0.66
Mondego Estuary (M)	6.4 ± 3.6	7.2 ± 5.9	14.0. ± 1.4	1.5	0.29
Venice Lagoon (V)	12.0 ± 1.2	11.2 ± 3.2	42.7 ± 8.4	15.7	0.001

For all treatments, *n*=4 in each site, with the exception of Urias and Mondego estuaries where *n*=3. Significant differences within a site across nutrient treatments are indicated by *F* and *P* values from the results of one-way anova.

In the Waquoit Bay subestuaries, where we measured ambient growth responses in different times of the year, we found a seasonal growth response of *U. lactuca* (Table 2). Growth was higher in summer than fall in all subestuaries, with July being the peak time of growth of *U. lactuca* (Table 2).

**Fig. 4:**
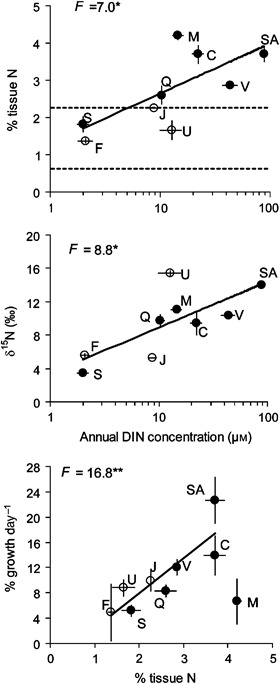
Percent tissue N (top) and *δ*^15^N (middle) of macroalgal fronds vs. annual dissolved inorganic nitrogen (DIN) concentrations, and growth rate vs. % tissue N (bottom) in the experimental sites. Dotted lines (top) refer to maximum and minimum N requirements for growth (Pedersen & Borum, 1996). Sites include Waquoit Bay, USA (S, Q, C), Venice Lagoon, Italy (V), Mondego Estuary, Portugal (M), Urias Estuary, Mexico (U), Jobos Bay, Puerto Rico (J), Flamengo Sound, Brazil (F), and San Antonio Bay, Argentina (SA). Temperate sites are black symbols and tropical sites are open symbols. Results of regression analyses are indicated by the *F* values and significance level of ^*^P=0.05 and ^**^P=0.01 for all points. Data for Mondego Estuary (M) in (bottom) was excluded as an outlier from the regression line.

The internal N content in fronds differed among the study sites and increased logarithmically as annual DIN concentrations increased (Fig. 4, top). Macroalgal fronds from all sites were above the minimum nutrient requirements (lower dotted line), and macroalgae from more than half of the sites were higher than the N requirements reported for maximum growth rates of *Ulva* spp. (upper dotted line, Fig. 4, top). The growth response of *Ulva* spp. significantly increased with increasing % tissue N (Fig. 4, bottom), with the exception of fronds from Mondego Estuary, supporting the notion that growth is closely linked to both external nutrient supply and internal nutrient pools.

The *δ*^15^N of the macroalgae from the study sites differed, ranging from 4 to 16‰ and increased logarithmically with increasing annual DIN concentration (Fig. 4, middle). Macroalgae from sites with oligotrophic waters (Sage Lot Pond, Flemengo Sound, and Jobos Bay) had substantially lower *δ*^15^N values than those from sites with eutrophic waters (Fig. 4, middle).

### Relative response of macroalgae to nutrient enrichment

**Fig. 5:**
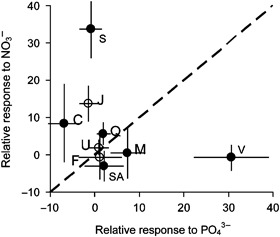
Relative response of *Ulva* spp. to NO_3_^−^ vs. PO_4_^3−^ enrichment. Relative response for each site was calculated as the difference between % growth day^−1^ in NO_3_^−^ or PO_4_^3−^ enriched treatments and the control treatments. Dotted line represents the 1 : 1 line (top). Sites include Waquoit Bay, USA (S, Q, C), Venice Lagoon, Italy (V), Mondego Estuary, Portugal (M), Urias Estuary, Mexico (U), Jobos Bay, Puerto Rico (J), Flamengo Sound, Brazil (F), and San Antonio Bay, Argentina (SA). Temperate sites are black symbols and tropical sites are open symbols (top).

**Fig. 6:**
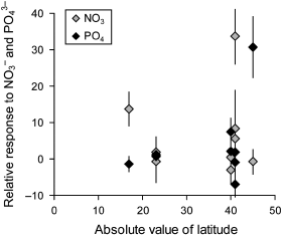
Relative response to NO_3_^−^ (gray diamonds) or PO_4_^3−^ (black diamonds) vs. absolute value of latitude.

There was a marked difference in relative response of *Ulva* spp. to nutrient enrichment in the different sites (Table 2; Fig. 5). Growth of *U. lactuca* in Sage Lot Pond, Jobos Bay, and Childs River was limited by the supply of NO_3_^−^, and, in contrast, the growth of *Ulva* spp. in Venice Lagoon, San Antonio Bay, and Mondego Estuary was restricted by supply of PO_4_^3−^ (Fig. 5). The other sites showed less conspicuous trends. The relative response to NO_3_^−^ and PO_4_^3−^, however, were not correlated to latitude (*F*=0.01, *P*=0.93 and *F*=1.1, *P*=0.32, respectively; Fig. 6). The magnitude of the relative response to either nutrient, however, was generally greater in higher latitudes (Fig. 6).

**Fig. 7:**
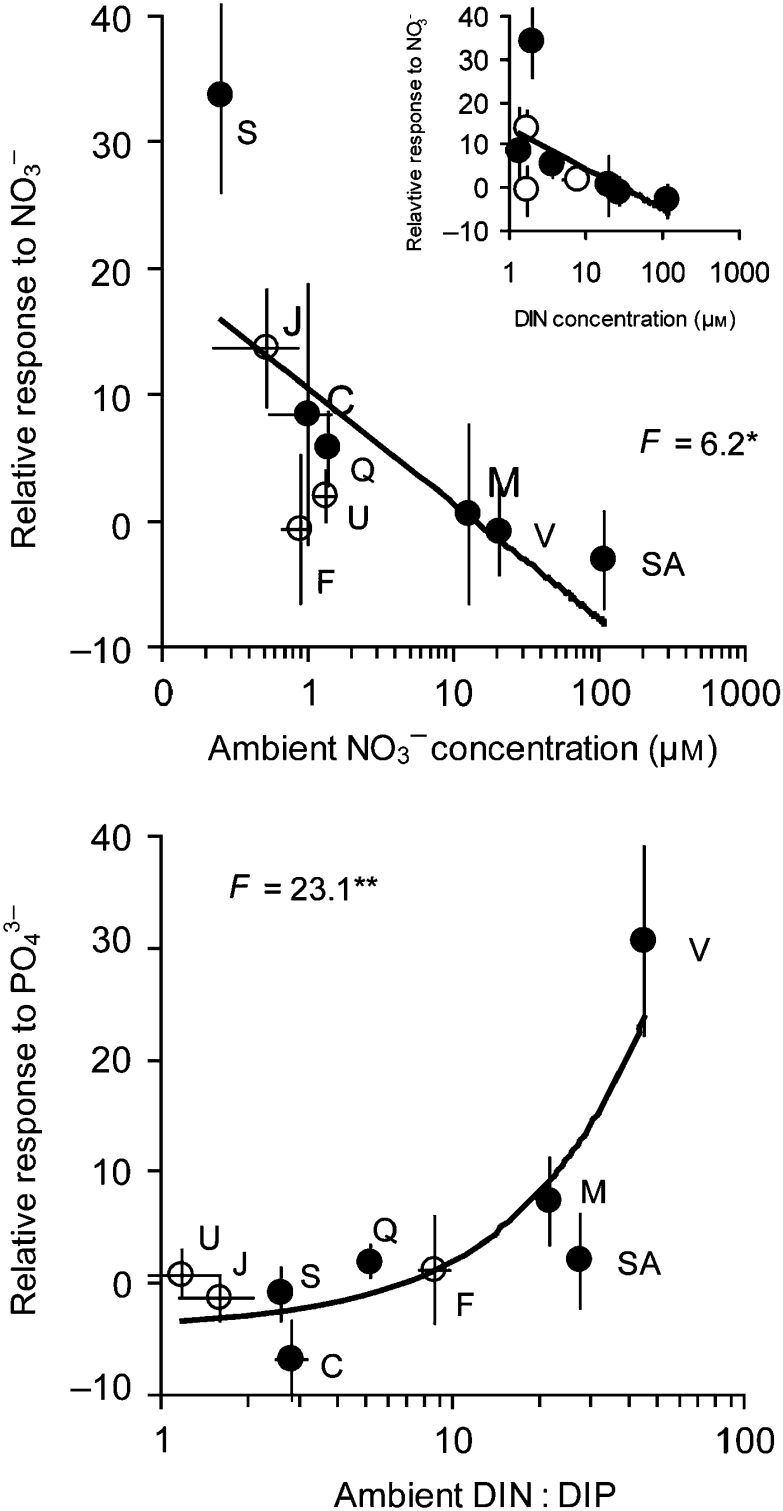
Relative response of *Ulva* spp. to NO_3_^−^ enrichment vs. ambient NO_3_^−^ concentration (top) and dissolved inorganic nitrogen (DIN) concentration (top; inset), and to PO_4_^3−^ enrichment vs. ambient DIN : DIP (bottom). Sites include Waquoit Bay, USA (S, Q, C), Venice Lagoon, Italy (V), Mondego Estuary, Portugal (M), Urias Estuary, Mexico (U), Jobos Bay, Puerto Rico (J), Flamengo Sound, Brazil (F), and San Antonio Bay, Argentina (SA). Temperate sites are black symbols and tropical sites are open symbols. Results of regression analyses are indicated by the *F* values and significance level of ^*^P=0.05 and ^**^P=0.01.

To ascertain whether the differences in responses to the nutrient enrichments were related to differences in ambient nutrient regimes in the different coastal waters, we plotted the growth response under enrichment vs. mean ambient nutrient concentrations in each site during the time of the experiments (Fig. 7). Relative response of fronds enriched with NO_3_^−^ decreased as ambient NO_3_^−^ increased (Fig. 7, top), demonstrating that fronds responded to NO_3_^−^ addition when NO_3_^−^ was in limited supply, and did not respond to NO_3_^−^ addition in environments where NO_3_^−^ was plentiful. We note that NO_3_^−^ was such a dominant contributor to the DIN that we could find a similar relationship of growth of *Ulva* spp. to DIN (Fig. 7, top, inset).

There was a significant increase in the relative growth of algal fronds to PO_4_^3−^ in relation to the ratio of DIN to DIP (Fig. 7, bottom). This relationship was largely a function of NO_3_^−^ supply, because growth of macroalgal fronds was not significantly related to PO_4_^3−^ concentration (data not shown). In Venice Lagoon, San Antonio Bay, and Mondego Estuary, where ambient DIN concentrations were highest, *Ulva* spp. increased growth in response to PO_4_^3−^ addition.

## Discussion

### Response of macroalgae to ambient conditions

This paper takes advantage of the presence of a model alga across many shores of the world, as well as a species that can be used in experimental manipulations, to provide evidence of the ecological response to a broad range of increasingly eutrophic conditions across the world.

The growth response of *Ulva* spp. was correlated to long-term ambient supply of nutrients in our study sites, suggesting that increased nutrient availability stimulates macroalgal growth. *Ulva* spp. in the control treatments were particularly driven by increases in annual DIN concentrations, but also responded to increased P supply. DIN rather than P, however, was in far greater supply in our study sites, as is the case in many coastal waters worldwide (Howarth, 2008). Our data suggest that increases in DIN concentrations lead to increased growth and abundance of macroalgae that could in turn lead to more frequent occurrences of macroalgal blooms.

The variability in growth rates in Waquoit Bay estuaries demonstrate that growth may differ throughout the growing season within a site, due to variability in ambient nutrient supply, temperature, and light intensity during the year. The variation in seasonal growth responses of *U. lactuca* in the subestuaries of Waquoit Bay is supported by studies on macroalgae in temperate sites (Peckol *et al.*, 1994; Pedersen & Borum, 1996; Balducci *et al.*, 2001). Thus, seasonality of growth responses must play a role in the occurrence of macroalgal blooms. Seasonality in responses, however, may be less marked in macroalgae from tropical sites, and blooms may be triggered by different factors, such as changes in freshwater inputs in wet season relative to dry season or cooler temperatures (Ochoa-Izaguirre *et al.*, 2002; Piñon-Gimate *et al.*, 2008), where shifts in algal assemblages may occur.

The internal N pools of macroalgal fronds were also linked to external DIN supply and were in the range of percent tissue N found in other studies of *Ulva* spp. (Fujita, 1985; Wheeler & Bjornsater, 1992; Pedersen & Borum, 1996; Lourenco *et al.*, 2005). All values of percent tissue N were above the critical N requirements for minimum growth, and fronds from some sites exceeded the requirements for maximum growth (Pedersen & Borum, 1996). Thus, percent tissue N values of *Ulva* spp. above 2.2% in this study likely represent excess N storage, which can reach up to 5.8% in *U. lactuca* (Pedersen & Borum, 1996). Judging from the size of internal N pools, growth of macroalgae in these waters may not be N-limited, or may be able to sustain maximum growth during times of lower nutrient availability. The ambient growth rates of *Ulva* spp. increased with internal N content, suggesting that both external and internal tissue N are strongly linked to growth and may be used as indicators of macroalgal growth response. Macroalgal growth rates in Mondego Estuary were an exception to this pattern, suggesting that growth may be limited by something other than nutrient supply. A recent study by Martins *et al.* (2007) showed through a spatially dynamic model that the productivity of green macroalgae varied considerably in Mondego Estuary, due to the hydrodynamics of the system and differences in temperature and light conditions at different depths of intertidal and subtidal areas. Therefore, it is important to consider that some systems may respond differently under varying environmental conditions. Despite these exceptions, it seems that growth of *Ulva* spp., and other fast growing algae, can be predicted by external and internal N history.

The range in *δ*^15^N of *Ulva* spp. from this study is similar to that of macroalgae from other studies exposed to heavy DIN sources (Table 3). These studies suggest that *δ*^15^N of macroalgae are heavier where DIN concentrations are higher. The heavy *δ*^15^N signatures of the fronds in our study sites were linked to high DIN concentrations, suggesting that the increased N loads were associated with wastewater rather than other N sources (McClelland & Valiela, 1998; Cole *et al.*, 2005), particularly so in Urias Estuary and San Antonio Bay. If fertilizer or atmospheric deposition was dominant in the sites where we did our study, *δ*^15^N of the fronds would have been near 0‰ (McClelland & Valiela, 1998). In many other case studies, the heaviest *δ*^15^N signatures were found in *Ulva* spp. compared with other macroalgal taxa (Table 3). Thus, *Ulva* spp. are a sensitive sentinel for detecting wastewater inputs to coastal waters, and might help predict the potential for future macroalgal blooms, because concentrations of wastewater-derived NO_3_^−^ in the water column determined *Ulva* spp. growth responses in our study sites. For the coastal waters we studied, therefore, the *δ*^15^N data suggest that wastewater was the major contributor to increases in DIN concentrations, and thus, likely to be the main cause of macroalgal blooms.

**Table 3:** Comparison of studies using macroalgal *δ*^15^N signatures as indicators of anthropogenic DIN inputs

Macroalgal taxa	Site	DIN concentration (μm)	Source of DIN	*δ*^15^N (‰)	Reference
*Ulva lactuca*	Narragansett Bay, RI, USA	2.7–130	Sewage effluent from treatment facilities	9–15	Pruell *et al.* (2006)
*Ulva* spp., *Ceramium* spp., *Polysiphonia* spp.,	Warnow River-system, Baltic Sea, NE Germany	5–265	Sewage effluent, manure	7.6–13.5 4.7–9.5 6.9–8.6	Deutsch & Voss (2006)
*Ulva lactuca*, *Chaetomorpha linum*, *Gracilaria tikvahiae*, *Caulerpa prolifera*,	East Central FL, USA	0.7–8.1	Treated sewage effluent and wastewater groundwater discharge	5–13	Barile (2004)
*Laurencia intricata*, *Cladophora catenata*, Other algae	Southern Florida Bay and Lower Florida Keys, USA	1–8.5	Sewage effluent	1–6.5 1–5.5 1–10	Lapointe *et al.* (2004)
*Fucus vesiculosus*	Himmerfjarden Bay, Sweden	21–32	Sewage effluent	3–9.5	Savage & Elmgren (2004)
*Ulva lactuca*, *Gracilaria tikvahiae*	Cape Cod estuaries MA, USA	2–12.6	Wastewater groundwater discharge	5–10	Cole *et al.* (2005)
*Ulva australus*, *Vidalia* spp., *Ecklonia radiata*	Ocean Reef, Western Australia	–	Sewage effluent	8.8–12.8 6.3–10.2 8–14	Gartner *et al.* (2002)
*Ulva* spp.	Boston Harbor MA, USA	10	Sewage effluent from treatment facilities	6.1–14.4	Tucker *et al.* (1999)

The range of *δ*^15^N correspond to distant from effluent source and/or range in DIN concentrations, with heavier *δ*^15^N being closer to the source and/or under higher DIN concentrations.

DIN, dissolved inorganic nitrogen.

### Relative response of macroalgae to nutrient enrichment

The relative growth response of *Ulva* spp. to experimental N or P enrichment varied across temperate and tropical sites and was not linked to geographic or latitudinal differences as had been previously suggested (Nixon & Pilson, 1983; Lapointe *et al.*, 1992; Howarth, 2008). Rather, we demonstrated that N and P limitation of growth was linked directly to nutrient availability. In the case of macroalgae of the genus *Ulva*, DIN supply, primarily in the form of NO_3_^−^, was a dominant controlling feature in all coastal waters studied, temperate or tropical: if DIN supply was low, it restricted macroalgal growth, and if DIN was high, PO_4_^3−^ became limiting. Although NO_3_^−^ has been previous found as a driving factor in macroalgal growth, no existing study before this study has simultaneously conducted experiments on macroalgal growth responses to nutrients on a global scale to test how widespread is this phenomenon.

The results of our study support the meta-analysis of Elser *et al.* (2007), which showed no consistent pattern in N or P limitation across latitudes. Although Elser *et al.* (2007) showed some correlation with N limitation being more common in temperate sites, and P limitation being more common in tropical sites, these correlations were very weak (*R*=0.22 and 0.34, respectively) and there were no references to ambient nutrient conditions of these studies sites, which could be more important than latitudinal differences alone.

We are unsure how general our findings may be for all macroalgal species, because we know that in macroalgae there are diverse, species-specific responses to nutrient supply (Fujita, 1985; Fong *et al.*, 2001; Teichberg *et al.*, 2008). *Ulva* spp., however, are rather widespread, and are the dominant protagonists of many instances of harmful macroalgal blooms across the world. It is probable that other fast-growing macroalgae show similar limitation in proportion to the ambient supply of these two nutrients in the water-column as *Ulva* spp. Barile (2004) found that in subtropical coastal waters of east-central Florida, where water N : P were on average 8 : 1, that macroalgae with high uptake affinities for DIN (*U. lactuca, Chaetomorpha linum, Enteromorpha intestinalis, Caulerpa* spp., and *Gracilaria tikvahiae*) were N limited. In Southeastern Brazil, growth of macroalgae was P limited where water N : P were greater than 16 : 1 (Lourenco *et al.*, 2005). In Waquoit Bay estuaries, where water N : P was approximately 3 : 1 during the growing season, *G. tikvahiae* were N limited (Teichberg *et al.*, 2008), along with *U. lactuca*, as reported in this study. Although there is variability in growth responses, uptake affinities, and tissue N and P storage capacities among macroalgal taxa (Fujita, 1985; Pedersen & Borum, 1996; Fong *et al.*, 2003), it is likely that water N : P may be useful to predict nutrient limitation in bloom forming species, as they were in our sites across the world. Thus, if the responses we report here are at all representative, the results have implications for many estuaries and coastal waters, tropical or temperate, where we are certain that human activities are increasing nutrient loads and changing N : P (Sfriso *et al.*, 1992; Valiela *et al.*, 1997; Larned, 1998; Lapointe *et al.*, 2004).

The results of this study demonstrate that nutrient supply strongly influenced growth and other features of *Ulva* spp., common macroalgae frequently involved in harmful macroalgal blooms. The comparisons of experimental results highlight the fact that as coastal waters increasingly undergo nutrient enrichment, blooms of algae will increase, and that the limiting element supporting the growth, initially nitrogen, shifts to phosphorus in those waters subject to the highest nitrogen loadings. It appears that understanding the specific mechanisms supporting macroalgal growth and managing the occurrence of blooms, regardless of temperate or tropical location, will require consideration of ambient supplies of N and P, as well as information on the magnitude of wastewater-related nutrient loads and the relative role of N and P in the specific locality involved. This result has additional applied relevance, because we have shown that ambient DIN concentrations and N : P are good predictors of macroalgal growth responses to nutrient enrichment.

Managers of coastal water quality are always facing the dilemma of not knowing explicitly what might be done to predict future time trends, maintain, or restore a coastal resource. If we know current loading rates, and we can predict future trajectory of nitrogen and phosphorus availability in a coastal water body, we might be able to use the information on N : P in water and macroalgal tissues to anticipate the formation of macroalgal blooms. In addition, knowing whether externally supplied nitrogen or phosphorus inputs might be restricting macroalgal growth may be a welcome asset, in particular because control of nitrogen is harder and more costly. These monitoring tools in combination with isotopic techniques may be used to detect the main nutrients responsible for bloom occurrences and point to specific sources to target for management.

## Supporting Information

**Table SA1.** Mean ambient water nutrientconcentrations ± standard error averaged from annual measurements from other studies and during the experiment incubation period in this study for each site.

## References

[b1] Aguiar Ab, Morgan JA, Teichberg M, Fox S, Valiela I (2003). Transplantation and isotopic evidence of the relative effects of ambient and internal nutrient supply on the growth of *Ulva lactuca*. Biological Bulletin.

[b2] Azevedo GFO (2002).

[b3] Baden SP, Loo LO, Pihl L, Rosenberg R (1990). Effects of eutrophication on benthic communities including fish: Swedish west coast. Ambio.

[b4] Balducci C, Sfriso A, Pavoni B (2001). Macrofauna impact on *Ulva rigida* C. Ag. production and relationship with environmental variables in the lagoon of Venice. Marine Environmental Research.

[b5] Barile PJ (2004). Evidence of anthropogenic nitrogen enrichment of the littoral waters of east central Florida. Journal of Coastal Research.

[b6] Bjornsater BR, Wheeler PA (1990). Effect of nitrogen and phosphorus supply on growth and tissue composition of *Ulva fenestrata* and *Enteromorpha intestinalis* (Ulvales, Chlorophyta). Journal of Phycology.

[b7] Bowen JL, Valiela I (2008). Using δ^15^N to assess coupling between watersheds and estuaries in temperate and tropical regions. Journal of Coastal Research.

[b8] Bricker SB, Longstaff B, Dennison W, Jones A, Boicourt K, Wicks C, Woerner J (2008). Effects of nutrient enrichment in the nation's estuaries: a decade of change. Harmful Algae.

[b9] Cardoso PG, Pardal MA, Lillebø AI, Ferreira SM, Raffaelli D, Marques JC (2004). Dynamic changes in seagrasses assemblages under eutrophication and implications for recovery. Journal of Experimental Marine Biology and Ecology.

[b10] Carrer GM, Todesco G, Bocci M, Lasserre P, Marzollo A (2000). Environmental monitoring in the Palude della Rosa, Lagoon of Venice. The Venice Lagoon Ecosystem: Inputs and Interactions between Land and Sea.

[b11] Cole ML, Kroeger KD, McClelland JW, Valiela I (2005). Macrophytes as indicators of land-derived wastewater: application of a δ^15^N method in aquatic systems. Water Resources Research.

[b12] Corbisier T, Soares L, Petti M, Muto E, Silva M, McClelland J, Valiela I (2006). Use of isotopic signatures to assess the food web in a tropical shallow marine ecosystem of Southeastern Brazil. Aquatic Ecology.

[b13] Cummins SP, Roberts DE, Zimmerman KD (2004). Effects of the green macroalga *Enteromorpha intestinalis* on macrobenthic and seagrass assemblages in a shallow coastal estuary. Marine Ecology Progress Series.

[b14] D'Avanzo C, Kremer JN, Wainright SC (1996). Ecosystem production and respiration in response to eutrophication in shallow temperate estuaries. Marine Ecology Progress Series.

[b15] Deutsch B, Voss B (2006). Anthropogenic nitrogen input traced by means of δ^15^N values in macroalgae: results from in-situ incubation experiments. Science of the Total Environment.

[b16] Díaz P, López Gappa JJ, Piriz ML (2002). Symptoms of eutrophication in intertidal macroalgal assemblages of Nuevo Gulf (Patagonia, Argentina). Botanica Marina.

[b17] Diaz RJ (2001). Overview of hypoxia around the world. Journal of Environmental Quality.

[b18] Duarte CM (1995). Submerged aquatic vegetation in relation to different nutrient regimes. Ophelia.

[b19] Elser JJ, Bracken MES, Cleland EE (2007). Global analysis of nitrogen and phosphorus limitation of primary producers in freshwater, marine, and terrestrial ecosystems. Ecology Letters.

[b20] Flindt M, Salomonsen J, Carrer M, Bocci M, Kamp-Nielsen L (1997). Loss, growth and transport dynamics of *Chaetomorpha aerea* and *Ulva rigida* in the Lagoon of Venice during early summer field campaign. Ecological Modelling.

[b21] Fong P, Boyer KE, Desmond JS, Zedler JB (1996). Salinity stress, nitrogen competition, and facilitation: what controls seasonal succession of two opportunistic green macroalgae?. Journal of Experimental Marine Biology and Ecology.

[b22] Fong P, Boyer KE, Kamer K, Boyle KA (2003). Influence of initial tissue nutrient status of tropical marine algae on response to nitrogen and phosphorus additions. Marine Ecology Progress Series.

[b23] Fong P, Kamer K, Boyer KE, Boyle KA (2001). Nutrient content of macroalgae with differing morphologies may indicate sources of nutrients for tropical marine systems. Marine Ecology Progress Series.

[b24] Fox SE, Olsen YS, Teichberg M, Valiela I, Kennish M, Paerl H Controls acting on benthic macrophyte communities in a temperate and a tropical estuary. Coastal Lagoons: Critical Habitats of Environmental Change.

[b25] Fox SE, Stieve E, Valiela I, Hauxwell J, McClelland J (2008). Macrophyte abundance in Waquoit Bay: effects of land-derived nitrogen loads on seasonal and multi-year biomass patterns. Estuaries and Coasts.

[b26] Fox SE, Teichberg M, Olsen YS, Heffner L, Valiela I (2009). Restructuring of benthic communities in eutrophic estuaries: lower abundance of prey leads to trophic shifts from omnivory to grazing. Marine Ecology Progress Series.

[b27] Fujita RM (1985). The role of nitrogen status in regulating transient ammonium uptake and nitrogen storage by macroalgae. Journal of Experimental Marine Biology and Ecology.

[b28] Gartner A, Lavery P, Smit AJ (2002). Use of δ^15^N signatures of different functional forms of macroalgae and filter-feeders to reveal temporal and spatial patterns in sewage dispersal. Marine Ecology Progress Series.

[b29] Hauxwell J, Cebrian J, Furlong C, Valiela I (2001). Macroalgal canopies contribute to eelgrass (*Zostera marina*) decline in temperate estuarine ecosystems. Ecology.

[b30] Hayden HS, Blomster J, Maggs CA, Silva PC, Stanhope MJ, Walland RJ (2003). Linnaeus was right all along *Ulva* and*Enteromorpha* are not distinct genera. European Journal of Phycology.

[b31] Holmes GT (2008).

[b32] Howarth RW (2008). Coastal nitrogen pollution: a review of sources and trends globally and regionally. Harmful Algae.

[b33] Howarth RW, Anderson D, Cloern J (2000). Nutrient pollution of coastal rivers, bays, and seas. Issues in Ecology.

[b117] Kamer K, Boyle KA, Fong P (2001). Macroalgal bloom dynamics in a highly eutrophic Southern California estuary. Estuaries.

[b35] Lapointe BE, Barile PJ, Littler MM, Littler DS (2005). Macroalgal blooms on southeast Florida coral reefs II. Cross-shelf discrimination of nitrogen sources indicates widespread assimilation of sewage nitrogen. Harmful Algae.

[b36] Lapointe BE, Barile PJ, Matzie WR (2004). Anthropogenic nutrient enrichment of seagrass and coral reef communities in the lower Florida keys: discrimination of local versus regional nitrogen sources. Journal of Experimental Marine Biology and Ecology.

[b37] Lapointe BE, Littler MM, Littler DS (1992). Nutrient availability to marine macroalgae in siliciclastic versus carbonate-rich coastal waters. Estuaries.

[b38] Lapointe BE, O'Connell J (1989). Nutrient-enhanced growth of *Cladophora prolifera* in Harrington Sound, Bermuda: eutrophication of a confined, phosphorus-limited marine ecosystem. Estuarine, Coastal and Shelf Science.

[b39] Larned ST (1998). Nitrogen- versus phosphorus-limited growth and sources of nutrients for coral reef macroalgae. Marine Biology.

[b40] Lavery PS, Lukatelich RJ, McComb AJ (1991). Changes in the biomass and species composition of macroalgae in a eutrophic estuary. Estuarine, Coastal and Shelf Science.

[b41] Lillebø AI, Neto JM, Martins I (2005). Management of a shallow temperate estuary to control eutrophication: the effect of hydrodynamics on the system's nutrient loading. Estuarine, Coastal and Shelf Science.

[b42] Liu D, Bai J, Song S, Zhang J, Sun P, Li Y, Han G (2007). The impact of sewage discharge on the macroalgal community in the Yellow Sea coastal area around Quingdao, China. Water, Air, and Soil Pollution: Focus.

[b43] Liu D, Keesing JK, Xing Q, Shi P (2009). World's largest macroalgal bloom caused by expansion of seaweed aquaculture in China. Marine Pollution Bulletin.

[b44] Lotze HK, Worm B, Sommer U (2000). Propagule banks, herbivory and nutrient supply control population development and dominance patterns in macroalgal blooms. Oikos.

[b45] Lourenco S, Barbarino E, Nascimento A, Paranhos R (2005). Seasonal variations in tissue nitrogen and phosphorus of eight macroalgae from a tropical hypersaline coastal environment. Cryptogamie Algologie.

[b46] Martins I, Lopes RJ, Lillebø AI, Neto JM, Pardal MA, Ferreira JG, Marques JC (2007). Significant variations in the productivity of green macroalgae in a mesotidal estuary: implications to the nutrient loading of the system and the adjacent coastal area. Marine Pollution Bulletin.

[b47] Martins I, Pardal M, Lillebo AI, Flindt MR, Marques JC (2001). Hydrodynamics as a major factor controlling the occurrence of green macroalgal blooms in a eutrophic estuary: a case study on the influence of precipitation and river management. Estuarine, Coastal and Shelf Science.

[b48] McClelland JW, Valiela I (1998). Linking nitrogen in estuarine producers to land-derived sources. Limnology and Oceanography.

[b49] McGlathery KJ, Marino R, Howarth RW (1994). Variable rates of phosphate-uptake by shallow marine carbonate sediments-mechanisms and ecological significance. Biogeochemistry.

[b50] Morand P, Merceron M (2005). Macroalgal population and sustainability. Journal of Coastal Research.

[b51] National Oceanic and Atmospheric Administration, Office of Ocean and Coastal Resource Management, National Estuarine Research Reserve System-Wide Monitoring Program (2004). http://cdmo.baruch.sc.edu.

[b52] National Research Council (NRC) (2000). Clean Coastal Waters: Understanding and Reducing the Effects of Nutrient Pollution.

[b53] Nixon SW, Oviatt C, Frithsen J, Sullivan B (1986). Nutrients and the productivity of estuarine and coastal marine ecosystems. Journal of the Limnological Society of Southern Africa.

[b54] Nixon SW, Pilson MEQ, Carpenter EJ, Capone DG (1983). Nitrogen in estuarine and coastal marine ecosystems. Nitrogen in the Marine Environment.

[b55] Ochoa-Izaguirre MJ, Carballo JL, Paez-Osuna F (2002). Qualitative changes in macroalgal assemblages under two contrasting climatic conditions in a subtropical estuary. Botanica Marina.

[b56] Oesterling M, Pihl L (2001). Effects of filamentous green algal mats on benthic macrofaunal functional feeding groups. Journal of Experimental Marine Biology and Ecology.

[b57] Oviatt C, Doering P, Nowicki B, Reed L, Cole J, Frithsen J (1995). An ecosystem level experiment on nutrient limitation in temperate coastal marine environments. Marine Ecology Progress Series.

[b58] Peckol P, DeMeo-Anderson B, Rivers J, Valiela I (1994). Growth, nutrient uptake capacities and tissue constituents of the macroalgae *Cladophora vagabunda* and *Gracilaria tikvahiae* related to site-specific nitrogen loading rates. Marine Biology.

[b59] Pedersen MF, Borum J (1996). Nutrient control of algal growth in estuarine waters. Nutrient limitation and the importance of nitrogen requirements and nitrogen storage among phytoplankton and species of macroalgae. Marine Ecology Progress Series.

[b60] Piñon-Gimate A, Serviere-Zaragoza E, Ochoa-Izaguirre MJ, Páez-Osuna F (2008). Species composition and seasonal changes in macroalgal blooms in lagoons along the southeastern Gulf of California. Botanica Marina.

[b61] Piriz ML, Eyras MC, Rostagno CM (2003). Changes in biomass and botanical composition of beach-cast seaweeds in a disturbed coastal area from Argentine Patagonia. Journal of Applied Phycology.

[b62] Pregnall AM, Miller SL (1988). Flux of ammonium from surf-zone and nearshore sediments in Nahant Bay, Massachusetts, USA in relation to free-living *Pilayella littoralis*. Marine Ecology Progress Series.

[b63] Pruell RJ, Taplin BK, Lake JL, Jayaraman S (2006). Nitrogen isotope ratios in estuarine biota collected along a nutrient gradient in Narragansett Bay, Rhode Island, USA. Marine Pollution Bulletin.

[b64] Raffaelli DG, Raven JA, Poole LJ (1998). Ecological impact of green macroalgal blooms. Oceanography and Marine Biology: An Annual Review.

[b65] Rosenberg R, Elmgren R, Fleischer S, Jonsson P, Persson G, Dahlin H (1990). Marine eutrophication case studies in Sweden. Ambio.

[b66] Savage C, Elmgren R (2004). Macroalgal (*Fucus vesiculosus*) δ^15^N values trace decrease in sewage influence. Ecological Applications.

[b67] Sfriso A, Marcomini A (1997). Macrophyte production in a shallow coastal lagoon: part II. Coupling with chemico-physical parameters and nutrient concentrations in waters. Marine Environmental Research.

[b68] Sfriso A, Pavoni B, Marcomini A, Orio AA (1992). Macroalgae, nutrient cycles, and pollutants in the Lagoon of Venice. Estuaries.

[b69] Smith SV (1981). Kanehoe Bay sewage diversion experiment: perspectives on ecosystem responses to nutritional perturbation. Pacific Sciences.

[b70] Smith VH, Schindler DW (2009). Eutrophication science: where do we go from here?. Trends in Ecology and Evolution.

[b71] Sugimoto K, Hiraoka K, Ohta S, Niimura Y, Terawaki T, Okada M (2007). Effects of ulvoid (*Ulva* spp.) accumulation on the structure and function of eelgrass (*Zostera marina* L.) bed. Marine Pollution Bulletin.

[b72] Teichberg M (2007).

[b73] Teichberg M, Fox SE, Aguila C, Olsen YS, Valiela I (2008). Macroalgal response to experimental nutrient enrichment in shallow coastal waters: growth, internal nutrient pools, and isotopic signatures. Marine Ecology Progress Series.

[b74] Teichberg M, Heffner L, Fox S, Valiela I (2007). Nitrate reductase and glutamine synthetase activity, internal N pools, and growth of *Ulva lactuca*: responses to long and short-term N supply. Marine Biology.

[b75] Thomsen MS, McGlathery KJ, Tyler AC (2006). Macroalgal distribution patterns in a shallow, soft-bottom lagoon, with emphasis on the nonnative *Gracilaria vermiculophylla* and *Codium fragile*. Estuaries and Coasts.

[b76] Thornber CS, DiMilla P, Nixon SW, McKinney RA (2008). Natural and anthropogenic nitrogen uptake by bloom-forming macroalgae. Marine Pollution Bulletin.

[b77] Tucker J, Sheats N, Giblin AE, Hopkinson CS, Montoya JP (1999). Using stable isotopes to trace sewage-derived material through Boston Harbor and Massachusetts Bay. Marine Environmental Research.

[b78] Valiela I (2006). Global Coastal Change.

[b79] Valiela I, Foreman K, LaMontagne M (1992). Couplings of watersheds and coastal waters- sources and consequences of nutrient enrichment in Waquoit Bay, Massachusetts. Estuaries.

[b80] Valiela I, McClelland J, Hauxwell J, Behr PJ, Hersh D, Foreman K (1997). Macroalgal blooms in shallow estuaries: controls and ecophysiological and ecosystem consequences. Limnology and Oceanography.

[b81] Wheeler PA, Bjornsater BR (1992). Seasonal fluctuations in tissue nitrogen, phosphorus, and N:P for 5 macroalgal species common to the Pacific-Northwest coast. Journal of Phycology.

[b82] Wilce RT, Schneider CW, Quinlan AV, Vanden Bosch K (1982). The life history and morphology of free-living *Pilayella littoralis* (L.) Kjellm. (Ectocarpaceae, Ectocarpales) in Nahant Bay, Massachusetts. Phycologia.

[b83] Worm B, Lotze HK, Bostrom C, Engkvist R, Labanauskas V, Sommer U (1999). Marine diversity shift linked to interactions among grazers, nutrients, and propagule banks. Marine Ecology Progress Series.

