# Supplementary material for: Eutrophication and macroalgal blooms in temperate and tropical coastal waters: nutrient enrichment experiments with Ulva spp
Source: Glob Chang Biol. 2010 Sep;16(9):2624–37. doi: 10.1111/j.1365-2486.2009.02108.x (PMC3627300; doi:10.1111/j.1365-2486.2009.02108.x)
Supplement: Supplementary file 1 [file gcb0016-2624-SD1.doc]

**Supporting information**

Appendix Table 1. Mean ambient water nutrient concentrations ± standard error averaged from annual measurements from other studies and during the experiment incubation period in this study for each site.

|  | Mean ambient water nutrient concentrations (μM) | | | | | | | | | |
| --- | --- | --- | --- | --- | --- | --- | --- | --- | --- | --- |
|  | Annual | | | |  | During incubation (this study) | | | | |
| Site | NO_3_^-^ | NH_4_^+^ | PO_4_^3-^ | DIN | Source | NO_3_^-^ | NH_4_^+^ | PO_4_^3-^ | DIN | DIN/DIP |
| Sage Lot Pond (S) | 0.4 ± 0.1 | 1.6 ± 0.2 | 0.23 ± 0.05 | 2.0 ± 0.2 | Holmes 2008 | 0.3 ± 0.02 | 1.7 ± 0.2 | 0.8 ± 0.03 | 2.0 ± 0.2 | 2.6 ± 0.1 |
|  |  |  |  |  |  |  |  |  |  |  |
| Flamengo Sound (F) | 1.1 ± 0.3 | 1.0 ± 0.1 | 0.33 ± 0.03 | 2.1 ± 0.4 | Azevedo 2002 | 0.9 ± 0.2 | 0.8 ± 0.05 | 0.2 ± 0.01 | 1.7 ± 0.2 | 8.7 ± 1.0 |
|  |  |  |  |  |  |  |  |  |  |  |
| Jobos Bay (J) | 0.11 ± 0.05 | 8.7 ± 0.6 | 0.69 ± 0.06 | 8.8 ± 0.6 | NOAA 2004 | 0.5 ± 0.3 | 1.2 ± 0.4 | 1.1 ± 0.4 | 1.7 ± 0.5 | 1.6 ± 0.5 |
|  |  |  |  |  |  |  |  |  |  |  |
| Quashnet River (Q) | 8.0 ± 0.8 | 2.3 ± 0.3 | 0.23 ± 0.03 | 10.3 ± 0.8 | Holmes 2008 | 1.4 ± 0.03 | 2.3 ± 0.4 | 0.7 ± 0.0 | 3.7 ±0.4 | 5.3 ± 0.1 |
|  |  |  |  |  |  |  |  |  |  |  |
| Urias Estuary (U) | 2.2 ± 1.0 | 10.6 ± 2.7 | 1.5 ± 0.6 | 12.8 ± 2.9 | Ochoa-Izaguirre et al. 2002 | 1.3 ± 0.2 | 6.7 ± 2.8 | 6.8 ± 0.8 | 8.0 ± 2.8 | 1.2 ± 0.4 |
|  |  |  |  |  |  |  |  |  |  |  |
| Mondego River (M) | 7.4 ± 1.4 | 7.2 ± 0.6 | 1.4 ± 0.1 | 14.6 ± 1.5 | Lillebø et al. 2005 | 12.9 ± 2.4 | 6.5 ± 0.6 | 0.90 ± 0.03 | 19.4 ±2.5 | 21.4 ± 0.1 |
|  |  |  |  |  |  |  |  |  |  |  |
| Childs River (C) | 20.6 ± 2.5 | 1.6 ± 0.2 | 0.22 ± 0.03 | 22.2 ± 2.5 | Holmes 2008 | 1.0 ± 0.4 | 0.4 ± 0.1 | 0.9 ± 0.03 | 1.4 ± 0.4 | 2.8 ± 0.3 |
|  |  |  |  |  |  |  |  |  |  |  |
| Palude della Rosa,  Venice Lagoon (V) | 34.6 ± 6.2 | 8.9 ± 0.8 | 0.7 ± 0.1 | 43.5 ± 6.2 | Carrer et al. 2000 | 20.7 ± 1.7 | 7.2 ± 0.7 | 0.6 ± 0.02 | 27.9 ± 1.9 | 45.6 ± 0.1 |
|  |  |  |  |  |  |  |  |  |  |  |
| San Antonio Bay (SA) | 79.8 ± 4.7 | 8.7 ± 3.1 | 14.8 ± 4.8 | 88.6 ±  5.6 | P. Martinetto unpubl. data | 110 ± 4 | 11.0 ± 2.9 | 4.4 ± 0.8 | 121 ± 5 | 27.4 ± 0.2 |

**References**

Azevedo GFO (2002) *Variabilidade sazonal (outono-primavera) da produtividade primária e biomassa fitoplantônica na Enseada do Flamengo, Ubatuba, Litoral Norte do Estado de São Paulo.* Thesis, Instituto Oceanográfico, Universidade de São Paulo, BRASIL.

Carrer GM, Todesco G, Bocci M (2000) Environmental monitoring in the Palude della Rosa, Lagoon of Venice. In: *The Venice Lagoon Ecosystem: Inputs and Interactions Between Land and Sea* (eds Lasserre P, Marzollo A) UNESCO and Parthenon Publishing Group, Paris.

Holmes GT (2008) *Nutrient supply, water residence time, temperature, and grazing as controls of size-fractionated phytoplankton biomass in shallow temperate estuarine ecosystems*. Ph D thesis, Boston University, Boston.

Lillebø AI, Neto JM, Martins I, Verdelhos T, Leston S, Cardoso PG, Ferreira SM, Marques JC, Pardal MA (2005) Management of a shallow temperate estuary to control eutrophication: The effect of hydrodynamics on the system’s nutrient loading. *Estuarine, Coastal and Shelf Science*, **65,** 697-707.

National Oceanic and Atmospheric Administration, Office of Ocean and Coastal Resource Management, National Estuarine Research Reserve System-wide Monitoring Program (2004) Centralized Data Management Office, Baruch Marine Field Lab, University of South Carolina, USA. http://cdmo.baruch.sc.edu.

Ochoa-Izaguirre MJ, Carballo JL, Paez-Osuna F (2002) Qualitative changes in macroalgal assemblages under two contrasting climatic conditions in a subtropical estuary. *Botanica Marina*, **45**, 130-138.
